# Anaesthetic management for endobronchial valve insertion: lessons learned from a single centre retrospective series and a literature review

**DOI:** 10.1186/s12871-018-0670-x

**Published:** 2018-12-27

**Authors:** Venkatesan Thiruvenkatarajan, Thomas Maycock, Dion Grosser, John Currie

**Affiliations:** 1 The Queen Elizabeth Hospital, Woodville, South Australia 5011 Australia; 2 The University of Adelaide, Adelaide, South Australia Australia; 3 Department of Anaesthesia, The Queen Elizabeth Hospital, Woodville, South Australia 5011 Australia; 4 Department of Respiratory Medicine, The Queen Elizabeth Hospital, Woodville, South Australia 5011 Australia

**Keywords:** Endobronchial valve, General anaesthesia, Sedation, Monitored care, Emphysema

## Abstract

**Background:**

Endoscopic lung volume reduction using one or more endobronchial valves is a treatment option for a select group of patients with severe emphysema. Patients presenting for this procedure pose various challenges to the anaesthetist; in addition to their lung condition, they are often elderly with multiple comorbidities. The procedure is usually performed outside the operating room. Monitored anaesthesia care with intravenous sedation, and general anaesthesia with an endotracheal tube have both been described for these procedures, aiming for adequate ventilation and haemodynamic stability.

**Methods:**

We present our experience on 20 of these procedures in relation to the anaesthetic techniques employed and discuss the perioperative challenges involved in managing these cases.

**Results:**

Twenty one planned endobronchial valve insertion procedures were identified on 18 patients. There were ten cases of monitored anaesthesia care with sedation and 10 cases which used general anaesthesia with an endotracheal tube. Two have been excluded; one had features of anaphylaxis and the procedure was abandoned, and the other required conversion from monitored anaesthesia care to general anaesthesia with endotracheal tube.

**Conclusions:**

Both monitored anaesthesia care with sedation and general anaesthesia with endotracheal tube were well tolerated during endobronchial valve insertion procedures. General anaesthesia with endotracheal tube may offer better interventional conditions, patient comfort and reduced anaesthetic time.

## Background

**Fig. 1:**
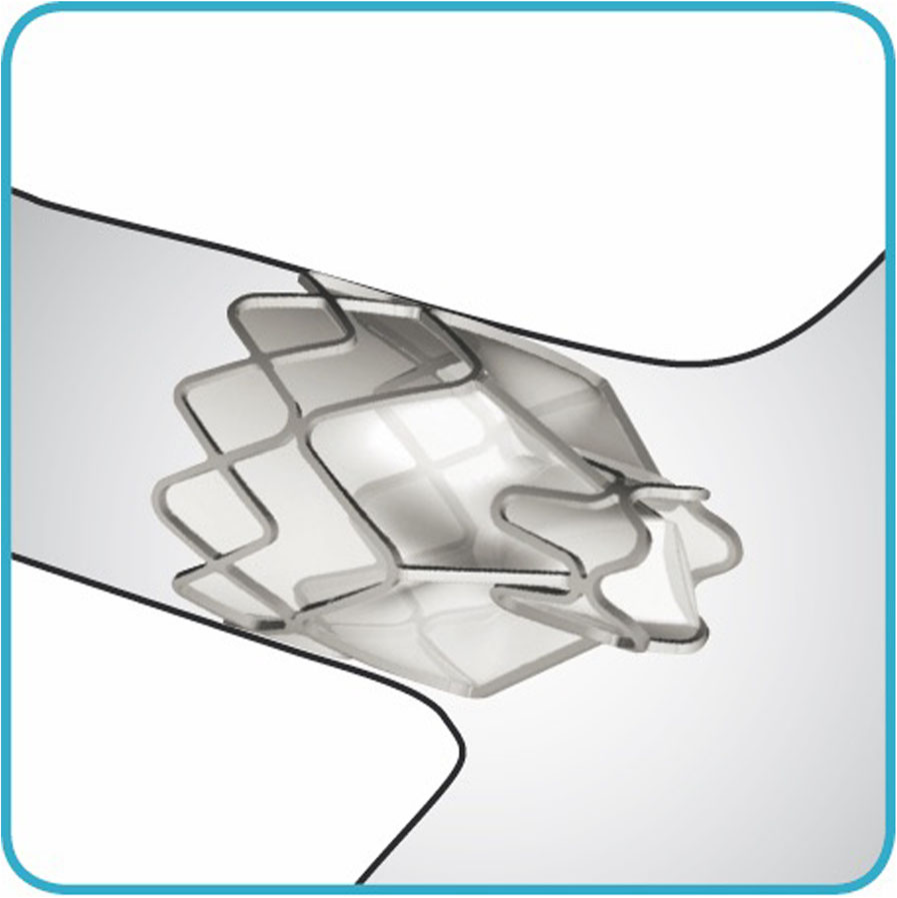
*Zephyr* endobronchial valve *insitu*

**Fig. 2:**
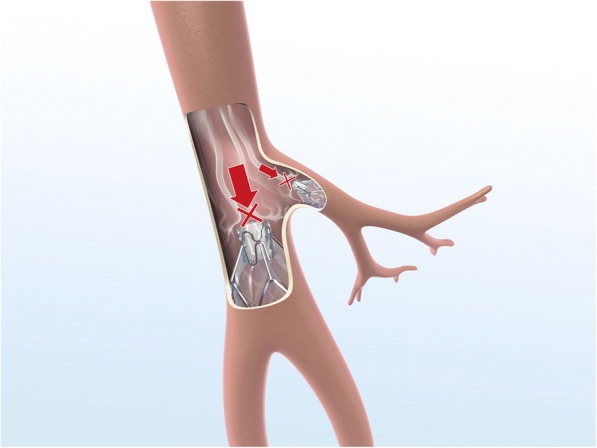
Flow through the valve during inspiration

**Fig. 3:**
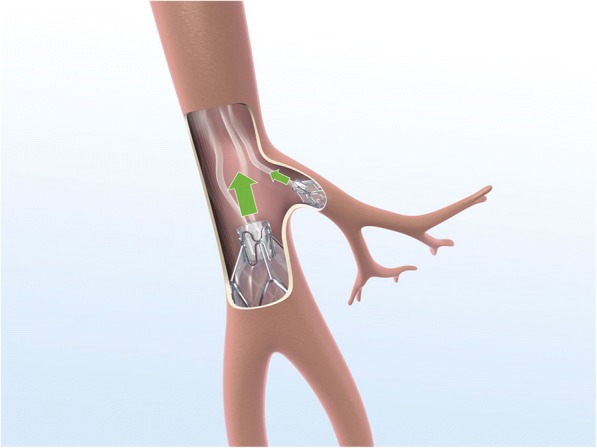
Flow through the valve during expiration

Endoscopic lung volume reduction (ELVR) using an endobronchial valve (EBV) is a treatment option for selected patients with severe emphysema [1, 2]. EBV uses a fibreoptic bronchoscope to implant one or more one-way valves into the airways of the most emphysematous part of the lung [2, 3]. The valve blocks the inspired air from entering the target lobe but allows air and secretions to leave during expiration, leading to lobar collapse and hence diminishing gas trapping (Figs. 1, 2 and 3) [2, 3]. Decreasing the volume of the affected region may increase overall lung elasticity, and promote healthier regions to expand and function more effectively.

The advanced age, and concurrent comorbidities of the patients, and the frequent use of a “remote location” such as a bronchoscopy suite, provide many challenges for anesthetists managing these cases. The anaesthetic requirements are: to provide good interventional bronchoscopic conditions; to facilitate patient comfort and minimise movement (during sedation); to maintain adequate ventilation whilst avoiding hypercapnia, and preventing air trapping (during general anaesthesia); to maintain haemodynamic stability; to diagnose and manage acute perioperative complications; and (ideally) allow an early recovery. General anaesthesia with endotracheal intubation (GA via ETT), and monitored anaesthesia care (MAC) using sedation together with airway topicalisation have both been described for these procedures [3–6]. Nonetheless, EBV is still a novel technique, and literature discussing the anaesthetic implications of this procedure is limited. We describe the anaesthetic management of 20 procedures of EBV implantation in 17 patients as a retrospective review, with the objective of analysing the anaesthetic techniques and complications encountered during and after this procedure.

## Methods

After ethics committee approval from the Central Adelaide Local Health Network (Approval No: Q20170604), we performed a retrospective chart review (manual and electronic records) of patients who underwent EBV implantation procedures between April 2015 and December 2017 at The Queen Elizabeth Hospital, South Australia. Perioperative medical records and discharge summaries were analysed. Patients’ demographic profile including age, gender, weight, American Society of Anesthesiologists (ASA) physical status were recorded. Preoperative pulmonary function tests and arterial blood gas analyses during the procedure were also reviewed.

The following were also recorded: anaesthetic data during the procedure, including the type of anaesthesia administered (MAC or GA via ETT); details of airway topicalisation; medications used for sedation and general anaesthesia; methods to secure the airway; duration of the procedure; and total anaesthetic time. In addition, perioperative and postoperative complications relating to anaesthesia or the procedure, such as haemodynamic and respiratory instability (eg. pneumothorax), and length of hospital stay were also documented.

### Anaesthetic techniques

This is a new procedure and not frequently performed. It is not surprising that each anaesthetist employs their own favourite technique (and even this may change with the experience of previous cases). Broadly the techniques used in our institution fall into two groups; general anaesthesia with a tracheally intubated patient, and ‘monitored anaesthesia care’ with a sedated unintubated patient.

**Fig. 4:**
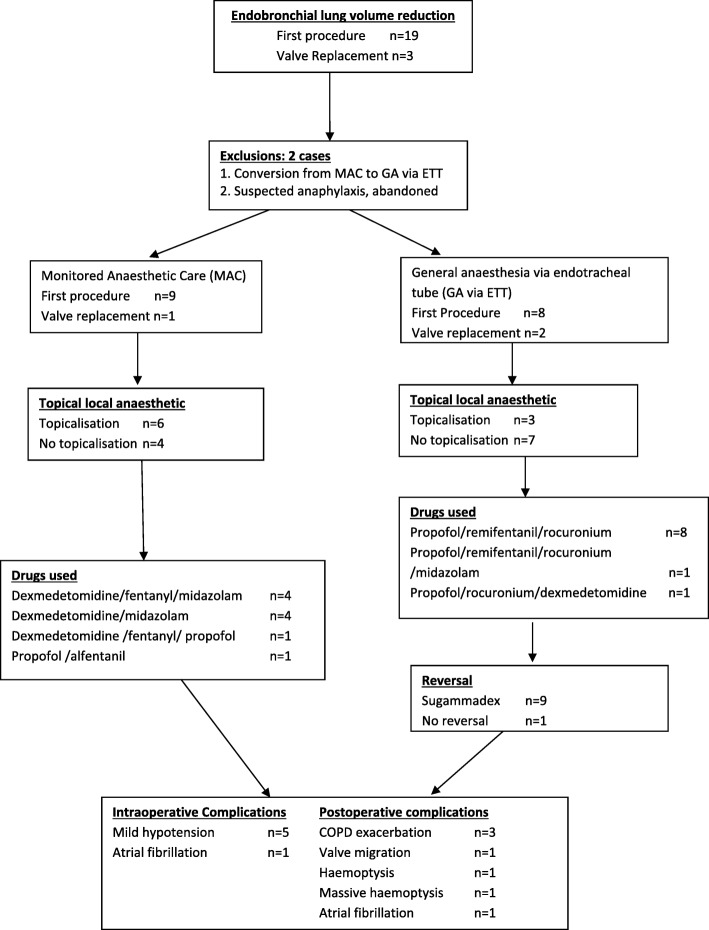
Flow chart detailing the cases retrieved, analysed, anaesthetic management and associated complications. COPD: chronic obstructive pulmonary disease

Dexmedetomidine using a bolus followed by infusion, was used in eight cases of MAC along with midazolam and fentanyl. In nine cases, glycopyrrolate was administered prior to commencing dexmedetomidine and six had airway topicalisation. Total intravenous anaesthesia (TIVA) with propofol was used in all GA via ETT cases. In 8 out of 10 of the GA via ETT cases, combinations of propofol, remifentanil and rocuronium were used. The details of the cases analysed presented in Fig. 4. Sugammadex was used as the reversal agent in 9 of the 10 GA cases. While the GA cases were positioned supine, MAC cases were positioned 15–20 degrees head up.

### EBV placement procedure

**Fig. 5:**
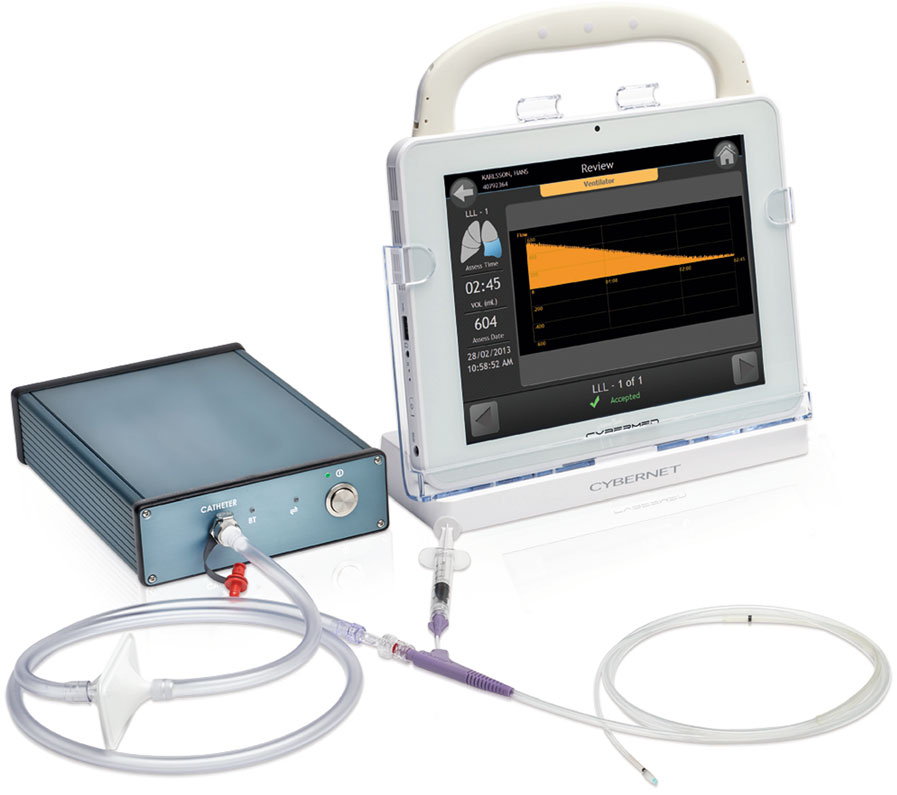
The Chartis console

**Fig. 6:**
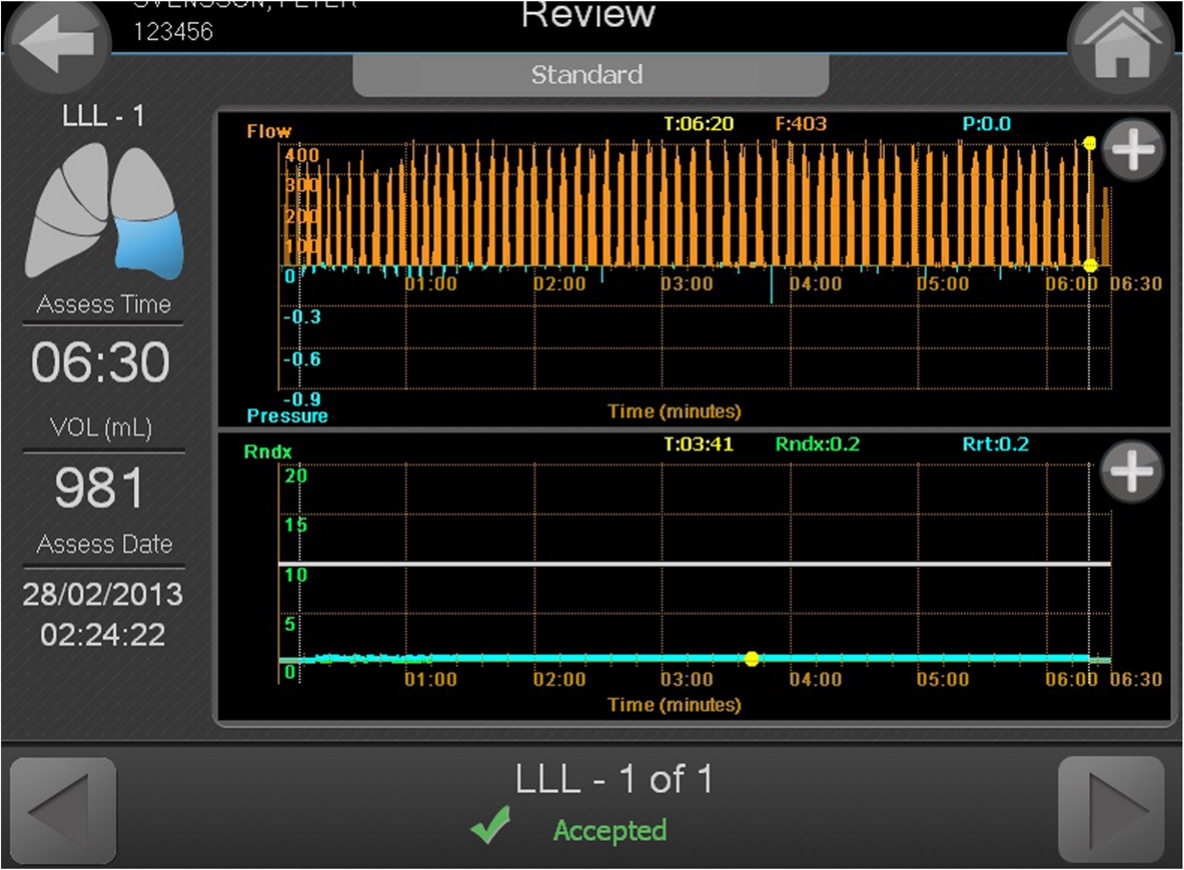
The Chartis console screen depicting assessments of time and volume of air exhaled from the target lobe. Example demonstrates presence of collateral ventilation during spontaneous ventilation

The technical aspects of this procedure from a pulmonology perspective have been extensively reviewed recently by two groups [1, 2]. The patients are selected according to generally agreed spirometry criteria, namely hyperinflation with a residual volume > 175% of predicted, and forced expiratory volume < 50% of predicted [1]. The efficacy of this technique depends on the absence of collateral ventilation between the lobe with implanted valves and the ipsilateral lobe [1]. Precise measurement of the amount of collateral ventilation is performed using endobronchial flow catheters. The Chartis Diagnostic System (Pulmonx, Redwood City, California, USA) is one such system [1]. This is an endobronchial flow catheter and associated computer software (Fig. 5). It is generally recommended that both the Chartis measurement and EBV implantation be performed as a single-stage procedure whenever possible, to minimize the risk of bronchoscopy induced COPD exacerbations [1]. The Chartis system comprises a balloon catheter attached to a sensor remotely housed in a console. The catheter is passed through the working channel of the bronchoscope; the main bronchus of the target lobe is blocked by inflating the balloon; and collateral ventilation is assessed by measuring the expiratory flow over time (Fig. 6). If no collateral flow is observed, a flexible delivery catheter is then used to deploy the EBV into the targeted bronchial lumen. An inbuilt measurement gauge in the catheter helps in choosing the appropriate size of the valve for the target lumen [7]. In our review, all the procedures were done by the same interventional pulmonologist, while different consultant anaesthetists participated in the anaesthetic management. An arterial line was inserted in all patients and serial blood gas analyses were performed.

## Results

A total of 22 EBV planned insertion procedures were identified on 19 patients. Three patients underwent a second procedure as a valve replacement technique. The procedure had to be abandoned in one patient who manifested severe haemodynamic instability mimicking an anaphylaxis. In one instance, there was a conversion from MAC to general anaesthesia. This patient had a refractory cough and the procedure could not be performed using MAC. (The case was excluded from the analysis). The mean preoperative FEV1 was 0.78 ± 0.26 (range: 0.43–1.62) litres and the mean residual volume was 178% of predicted ±23.49 (range: 143–212) (Table 1).

**Table 1:** Demographic characteristics and clinical features of 17 patients who underwent 20 endobronchial valve insertions

Clinical Presentation	
Age (years)	65.77 ± 8.40
Male/female	10/10
Indications	
Emphysema lung volume reduction	17
Emphysema lung volume reduction Replacement – valve replacement	3
Baseline spirometry	
FEV_1_^a^	
Actual FEV_1_ (litres)	0.78 ± 0.26 (0.43–1.62)
Percentage predicted	29.05 ± 9.27 (18–48)
Residual volume (RV)	
Actual residual volume (litres)^b^	3.66 ± 0.64 (2.79–5.17)
Percentage predicted^c^	178 ± 23.49 (143–212)
RV/Total lung capacity (RV/TLC)^d^	
Ratio %	58.94 ± 6.02 (51–64.90)
Percentage predicted	140 ± 30.76 (127–162)

Data are presented as mean ± SD (range)

^a^Data was available for 18 procedures; ^b^ Data was available for 11 procedures; ^c^ Data was available for 15 procedures; ^d^ Data was available for 10 procedures

All were ASA status III with multiple co-morbidities. Emphysema was the most common indication. Fifteen patients had the Chartis endobronchial evaluation for collateral ventilation performed immediately prior to the valve insertion. A Zephyr valve (Pulmonx) was used in all cases and the procedures were performed in a dedicated bronchoscopy suite located outside the main operating room complex. Although the average procedure duration was similar between the MAC (24.90 min) and GA via ETT (20.70 min) group, the total anaesthetic time was much longer for the MAC group (61 min vs 37 min). Of the analysed 20 patients, 10 patients had MAC and 10 had GA with ETT. All GA via ETT cases were extubated at the end of the procedure (Fig. 4).

**Fig. 7:**
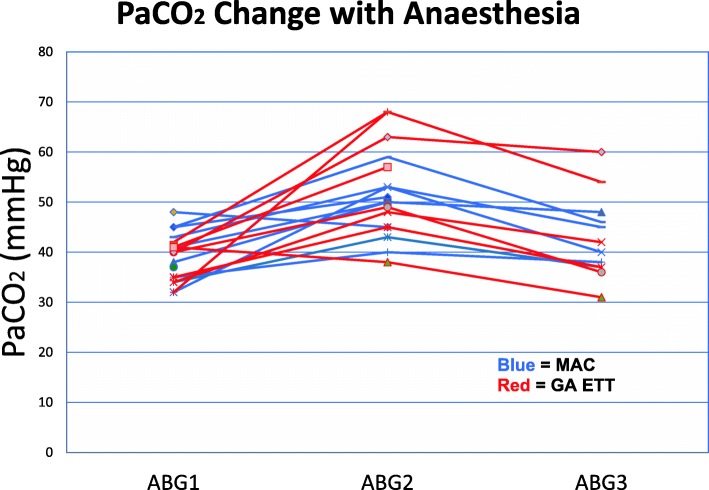
PaCO_2_ documented in this series of patients. ABG1 = Pre op, ABG2 = Intra op, ABG3 = 2 h post procedure. *All values are presented including incomplete data sets

All values of PaCO_2_ which were recorded in the patients’ case notes are shown in Fig. 7. The mean intraoperative rise in PaCO_2_ was 11 mmHg. By 2 h, the PaCO_2_ returned to near baseline values.

### Intraoperative complications

Mild hypotension was observed in 5 patients. The hypotension was treated with a single bolus of metaraminol in 4 cases and a bolus of ephedrine in 1 case. Intravenous infusions of inotropes or vasopressors were not required. One case of profound hypotension - thought likely to be anaphylaxis - occurred in the GA via ETT group, and the procedure was abandoned. There was one case of inadequate topicalisation. All patients were discharged to the respiratory high dependency unit.

### Postoperative complications

Postoperative complications were encountered in seven patients. One episode of rapid AF occurred on extubation. There were exacerbations of chronic obstructive pulmonary disease in three instances, and two cases of haemoptysis (one being described as massive) and one valve migration (Table 2). There were no pneumothoraces encountered and the mean hospital length of stay was 5 days. As an aid to comprehending the distribution of these complications, they are also included in Fig. 4.

**Table 2:** Intraoperative and postoperative complications

Intraoperative events	
Hypotension requiring vasopressors	5
AF with fast ventricular rate at extubation	1
Postoperative complications	
COPD exacerbation	2
Transient SVT (AF) and respiratory distress	1
COPD exacerbation and haemoptysis	1
Massive haempotysis	1
Valve migration	1

Data are presented as absolute numbers

*AF* Atrial fibrillation, *COPD* Chronic obstructive pulmonary disease, *SVT* Supraventricular tachycardia

## Discussion

Our review demonstrates that EBLV was well tolerated by this high-risk cohort of patients, with either MAC or GA via ETT and the peri-procedural complications were low. The PaCO_2_ trends were similar using both techniques. As a minimally invasive approach, endoscopic lung volume reduction (ELVR) has been shown to improve pulmonary function, exercise capacity, and quality of life in patients with severe emphysema [1]. Two types of valves are commercially available, the Zephyr valve (Pulmonx) and the Spiration valve (Olympus, Japan) [2]. The Zephyr valve is a second generation device that has replaced the first generation Emphasys valve, but the recent literature has been focused predominantly around the Zephyr valve (Fig. 1). Compared to the first generation valves, the Zephyr valves offer less flow resistance and promote better air flow especially with the larger valves. Also, the deployment manoeuvre is much simpler allowing them to be placed under lighter levels of anaesthesia [7].

While, an earlier case series on the first generation valve insertion described the application of GA via ETT [4] both MAC and GA via ETT have been described for the deployment of Zephyr valves as well for the Chartis measurement [3–6]. A recent recommendation by an expert panel on ELVR is in favour of advocating a GA via ETT technique. The arguments in its support are: a more secure airway; less distortion to the Chartis measurement - especially with low ventilation rates of 8–10/min and prolonged expiration (I/E ratio 1:3–1:4) [8]. Further, ventilation helps in reducing CO_2_ retention [1]. General anaesthesia also reduces the duration of the Chartis measurement [1].

Air trapping (increased residual volume and increased ratio of residual volume to total lung capacity) and development of intrinsic positive end-expiratory pressure (PEEPi) is a constant threat in patients with advanced COPD. There is elevated expiratory airway resistance, along with expiratory airflow limitation, reduced elastic recoil, and diminished expiratory time due to increased ventilatory rates. This impedes the pulmonary system in reaching the ideal elastic equilibration volume [9–11]. This phenomenon, referred to as dynamic hyperinflation can potentiate V/Q (ventilation/perfusion) mismatch, hypoxia, hypercapnia, barotrauma, and haemodynamic instability due to impaired venous return [12]. During EBLV procedures, ventilation is further compromised, as the respiratory passage is shared with bronchoscopy instruments, further limiting expiratory airflow. Ventilatory management should be targeted at reducing the dynamic hyperinflation, PEEPi, and air trapping [13]. Allowing a longer expiratory time (I/E ratio 1:3–1:4) is a major consideration to reducing air trapping and thereby decreasing the occurrence of ‘breath stacking’. Keeping a vigil on dynamic hyperinflation is paramount. One recommended way to differentiate dynamic hyperinflation from tension pneumothorax during GA is to detach the ventilator circuit from the endotracheal tube and observing for the blood pressure to return to baseline (usually within a minute). In our series, information as to the exact ventilatory pressures and I:E ratios employed was poorly documented. However, it is likely that appropriate and optimal ventilator settings would have been utilized.

### GA techniques

A TIVA technique may be preferable compared to a volatile based anaesthetic regime. Leakage of volatile agents is a concern due to repeated airway manipulation. Additionally, propofol may have a favourable effect of maintaining hypoxic pulmonary vasoconstriction, an effect beneficial in this scenario [14]. Remifentanil may be a good narcotic choice considering its potency and rapid onset and offset, allowing immediate return of respiratory function post procedure. Moreover, by continuing a low dose infusion during extubation, cough and hypertensive responses could be lessened. Sugammadex may be preferable to prevent residual neuromuscular blockade in the presence of compromised baseline respiratory function.

### Monitored Anaesthesia care

Monitored anaesthesia care with sedation along with airway topicalisation are guided by Institutional practices. Adequate airway topicalisation may help in preventing cough and movement. Mucosal atomizing devices such as the DeVilbiss (DeVilbiss health care, Devilbiss International), can generate local anaesthetic droplets between 6 and 12 μm, which are delivered to the distal tracheobronchial tree [15]. Excessive suctioning to clear secretions can result in airway oedema, mucosal bleeding and inflammation, and affect the valve sizing judgements [1]. Apart from reducing the secretions, an antisialagogue might also help in reducing the dilution of the delivered local anaesthetic into the tracheobronchial tree during airway topicalisation. However, antisialagogue benefits should be weighed against the risk of their chronotropic effects [1]. Achieving optimal sedation is always challenging, and never more so than during the Chartis Procedure. The patients’ should display adequate tidal volume to validate the detection of a collateral-free target lobe. This should be balanced against minimizing coughing and/or secretion production [1]. Avoiding respiratory failure and CO_2_ retention is a major concern during sedation techniques. Deep sedation should be attempted only if appropriate rescue resources are readily available [1]. Titrated doses of a short acting benzodiazepine and a narcotic may be an attractive option, as the effects can be readily reversed. Dexmedetomidine may be a promising selection in view of its sympatholytic and respiratory stability effects. The sympatholytic effect needs careful monitoring in these patients. For MAC technique, co–induction using reversible agents which reduce the requirements for dexmedetomidine, and thereby avoiding large drops in arterial pressure seemed popular with our anaesthetists.

An increasing trend towards increasing GA utilization was noted in our review. It became evident that GA via ETT enabled quicker Chartis measurements. The absence of cough and movement made the technique easier. A GA technique also meant that any prolonged procedure could be easily tolerated. An increase in the anaesthetic times of about 24 min were noted for the MAC group compared with the GA via ETT group (61 vs 37 min). Reassuring the patients, and gaining their cooperation takes time; likewise, obtaining effective topical anaesthesia of the airway further prolongs the anaesthetic time required. Currently, GA via ETT is the preferred choice at our institution. A supraglottic airway together with neuromuscular blockade may also offer similar bronchoscopic conditions without the need for intubation. However, the bronchoscope may be inserted and removed on more than one occasion, and trauma to the vocal cords and surrounding structures from repeated passage of the bronchoscope; along with the risk of displacement of a supraglottic device, means that an ETT may prove to be the preferred choice. Making such decisions must involve the pulmonologist, and close cooperation between the proceduralist and the anaesthetist is absolutely essential.

A wide range of complications can occur during the procedure, such as respiratory and also haemodynamic instability. None of the patients own medications (such as beta blockers and calcium channel blockers) were omitted preoperatively and there is a possibility of these agents worsening dexmedetomidine induced hypotension. This does not seem to have been a significant problem in our patients. The liberal use of glycopyrrolate prior to commencing dexmedetomidine may have averted significant bradycardia. The hypotension observed in the GA via ETT cases was in line with the expected drop after any general anaesthetic. It responded to boluses of vasopressors namely ephedrine and metaraminol, and no infusions (either vasopressors or inotropes) were required.

Pneumothorax is a major complication of EBV procedures, attributed to the sudden expansion of the lobe adjacent to the target lobe after the collapse of the treated lobe, and patients showing significant volume reduction on post procedure Chest X-ray may be at an increased risk [1]. The incidence varies between 15 and 25% [16], and the median onset is 2 days post procedure [16, 17]. It can also occur within hours after valve insertion and anytime during the procedure [2]. Two studies have looked into the factors responsible for pneumothorax occurrence after EBV insertion [17, 18]. One found that a strategy of post procedure bed rest and cough reduction helped decrease the incidence [18].

Presently, it is unclear whether the type of anaesthetic technique has any role in precipitating pneumothorax. Although, there is a theoretical possibility of increased pneumothorax with positive pressure ventilation in these high risk patients especially with bullous lung disease, the risk may be reduced if a specific individually optimised ventilatory approach is adhered to. If High Frequency Jet Ventilation is available this may be an attractive option, however, we do not have this device in our institution. If a small pneumothorax does occur, − in the absence of tension - careful monitoring should suffice. A chest drain is indicated for larger pneumothoraces. Infections, COPD exacerbations, pneumonia and temporary haemoptysis are other possible postoperative complications [2].

### Limitations of this review

The small numbers, the retrospective nature and missing data are some of the limitations of this review.

## Conclusion

To conclude, both MAC and GA via ETT were well tolerated during ELVR in a high risk group of patients with multiple comorbidities. On one hand, hypercapnia is a major threat during sedation, on the other, positive pressure ventilation may impose a pneumothorax risk. Nonetheless, GA via ETT strategy may offer superior interventional conditions, patient comfort and reduced anaesthetic time both during the Chartis measurement as well as during valve deployment. Close communication between the interventional pulmonologist and the anaesthetist is crucial in identifying and managing serious peri-procedural complications.

## Abbreviations

EBV: Endobronchial valve
ELVR: Endoscopic lung volume reduction
GA via ETT: General anaesthesia with endotracheal intubation
MAC: Monitored anaesthesia care with sedation
TIVA: Total intravenous anesthesia

## References

[1] Slebos DJ, Shah PL, Herth FJ, Valipour A (2017). Endobronchial valves for endoscopic lung volume reduction: best practice recommendations from expert panel on endoscopic lung volume reduction. Respiration.

[2] Jarad N (2016). Clinical review: endobronchial valve treatment for emphysema. Chron Respir Dis.

[3] Davey C, Zoumot Z, Jordan S, McNulty WH, Carr DH, Hind MD (2015). Bronchoscopic lung volume reduction with endobronchial valves for patients with heterogeneous emphysema and intact interlobar fissures (the BeLieVeR-HIFi study): a randomised controlled trial. Lancet.

[4] Hillier JE, Toma TP, Gillbe CE (2004). Bronchoscopic lung volume reduction in patients with severe emphysema: anesthetic management. Anesth Analg.

[5] Trudzinski FC, Höink AJ, Leppert D, Fähndrich S, Wilkens H, Graeter TP (2016). Endoscopic lung volume reduction using endobronchial valves in patients with severe emphysema and very low FEV1. Respiration.

[6] Cordovilla R, Torracchi AM, Novoa N, Jiménez M, Aranda JL, Varela G (2015). Endobronchial valves in the treatment of persistent air leak, an alternative to surgery. Arch Bronconeumol.

[7] One way valve for bronchoscopic lung volume reduction. http://www.ctsnet.org. Accessed 2 Sept 2018.

[8] Koster TD, van Rikxoort EM, Huebner RH, Doellinger F, Klooster K, Charbonnier JP (2016). Predicting lung volume reduction after endobronchial valve therapy is maximized using a combination of diagnostic tools. Respiration.

[9] Lumb A, Biercamp C (2014). Chronic obstructive pulmonary disease and anaesthesia. Contin Educ Anaesth Crit Care Pain.

[10] Rossi A, Ganassini A, Polese G, Grassi V (1997). Pulmonary hyperinflation and ventilator-dependent patients. Eur Respir J.

[11] Tobin MJ, Jubran A, Laghi F (2001). Patient-ventilator interaction. Am J Respir Crit Care Med.

[12] Yamakage M, Iwasaki S, Namiki A (2008). Guideline-oriented perioperative management of patients with bronchial asthma and chronic obstructive pulmonary disease. J Anesth.

[13] Duggappa DR, Rao GV, Kannan S (2015). Anaesthesia for patient with chronic obstructive pulmonary disease. Indian J Anaesth.

[14] Van Keer L, Van Aken H, Vandermeersch E, Vermaut G, Lerut T (1989). Propofol does not inhibit hypoxic pulmonary vasoconstriction in humans. J Clin Anesth.

[15] Chan KN, Clay MM, Silverman M (1990). Output characteristics of DeVilbiss no. 40 hand-held jet nebulizers. Eur Respir J.

[16] Gompelmann D, Herth FJ, Slebos DJ, Valipour A, Ernst A, Criner GJ (2014). Pneumothorax following endobronchial valve therapy and its impact on clinical outcomes in severe emphysema. Respiration.

[17] Valipour A, Slebos DJ, de Oliveira HG, Eberhardt R, Freitag L, Criner GJ (2014). Expert statement: pneumothorax associated with endoscopic valve therapy for emphysema--potential mechanisms, treatment algorithm, and case examples. Respiration.

[18] Herzog D, Poellinger A, Doellinger F, Schuermann D, Temmesfeld-Wollbrueck B, Froeling V (2015). Modifying post-operative medical care after EBV implant may reduce pneumothorax incidence. PLoS One.

